# Developing a competency scale for selecting and assessing psychological peer counselors in ethnic-area colleges: a pilot study in Guizhou Province, China

**DOI:** 10.3389/fpsyg.2024.1402403

**Published:** 2024-12-11

**Authors:** Junru Guo, Wei Chen, Meilan Liu, Lirong Jiang, Yurong Chen, Xiaoqing Zhao, Dan Peng, Xuefeng Jiang, Li Wang, Dongmei Wang

**Affiliations:** ^1^Department of Psychology, Guizhou Minzu University, Guiyang, China; ^2^School of Psychology, Guizhou Normal University, Guiyang, China; ^3^CAS Key Laboratory of Mental Health, Institute of Psychology, Chinese Academy of Sciences, Beijing, China; ^4^Maochang Middle School, Bijie, China; ^5^Student Affairs Office, Guizhou University, Guiyang, China; ^6^Student Affairs Office, Guizhou Medical University, Guiyang, China; ^7^Student Affairs Office, Dalian Minzu University, Dalian, China

**Keywords:** ethnic-area, college student, psychological peer counselors, competency characteristics, Chinese experience

## Abstract

**Introduction:**

Psychological peer counselors play a crucial role in the mental health education of college students, especially in ethnic regions. The study zeroes in on developing a tool for selecting and assessing psychological peer counselors in ethnic-area colleges and universities, using Guizhou Province as a case study.

**Materials and methods:**

Focusing on psychological peer counselors in ethnic-area higher education institutions, this study amalgamates open-ended questionnaire surveys, interviews, and literature analysis to construct a competency characteristics questionnaire. The questionnaire underwent pretesting with 450 psychological peer counselors in colleges and universities, involving entry analysis and exploratory factor analysis. Subsequently, it was administered formally to 570 psychological peer counselors for confirmatory factor analysis, coupled with internal consistency reliability tests at the empirical validity level. A subset of 230 psychological peer counselors was retested after three-month intervals. The Interpersonal Reaction Coefficient Scale (IRC-C) and the General Self-Efficacy Scale (GSES) were employed for validity tests.

**Results:**

The questionnaire consists of 21 items across four factors—role identity, communication sensitivity, personal traits, and professional ethic—cumulatively explaining 66.26% of the variance. The validated factor analyses demonstrated a good model fit (*X^2^/df* = 2.67, *SRMR* = 0.04, *TLI/NNFI* = 0.93, *CFI* = 0.94, *RMSEA* = 0.05, *AIC* = 21960.17, *BIC* = 22261.10). The questionnaire’s total and factor scores correlated positively with the total scores of the IRC-C/GSES (*r* = 0.18–0.64; all *p* < 0.01). The Cronbach’s alpha coefficients for the total questionnaire and individual factors ranged from 0.73 to 0.95, retest reliability from 0.64 to 0.92, and split-half reliabilities from 0.71 to 0.94.

**Conclusion:**

The Competency Scale for Psychological Peer Counselors in Colleges of Ethnic Areas demonstrates robust reliability and validity, making it a reliable tool for future screening of psychological peer counselors in ethnic-area colleges and universities.

## 1 Introduction

“Peer support” can be defined as a variety of interpersonal helping behaviors assumed by nonprofessionals who undertake a helping role with others who are “peers.” It can include one-to-one helping relationships, group leadership, advice, tutoring, and all activities of an interpersonal helping or assisting nature (Kracen et al., 2003). The psychological peer counselor refers to an individual selected from a group who, after receiving specific systematic training, provides psychological support and guidance to group members using basic psychological techniques, all while maintaining the role of a friend and sharing the same age group without hierarchical differences (Mamarchev, 1981). Peer relationships play a very important role in helping students cope with college life. For example, one study showed that 78% of students seek support from close friends when they feel worried or stressed, compared to 4% who seek help from school counselor (Moukaddem, 1995). Ning et al. conducted focus group interviews to investigate Chinese college students’ views on seeking mental health counseling. The results indicated that a lack of trust in counselors and the stigma of mental illness are major factors discouraging students from seeking professional help. However, peer influence can also create opportunities to challenge stigma and discrimination. When students share their positive counseling experiences with peers, it seems to reduce the stigma associated with mental illness and seeking help (Ning et al., 2022). There is evidence that peer support or “peer-led” programs in college can improve help-seeking behavior (Gravitz and Woods, 1976), increase social integration (Hixenbaugh et al., 2016), and reduce anxiety or depression scores (Byrom, 2018; Ellis et al., 2011; Fontana et al., 1999; Pfeiffer et al., 2011). In extreme cases of suicide, suicidal young people are more likely to talk about their problems with their peers than with their parents, teachers, or counselors (Abbey et al., 1989). In summary, college mental health peer supporters can initiate inexpensive supplemental campus wellness support programs that provide students with active listening, resource linking, and educating peers about self-care and healthy coping strategies (Caporale-Berkowitz, 2022; Johnson and Riley, 2021).

The mental health issues of ethnic minority college students have attracted widespread attention (Cheng et al., 2013; Gao et al., 2024; Vacek et al., 2011; Xin and Liu, 2019; Zeng et al., 2019). Research indicates disparities in physical and mental health between ethnic minority and other college students (Li and Liu, 2014; Wei et al., 2017). Ethnic minority college students in China exhibit a notably lower overall mental health level compared to the national average (Gao et al., 2013), with a higher prevalence of mental disorders, including obsessive-compulsive symptoms, anxiety, and depression (Luo et al., 2010; Wu, 2019). China, as a multi-ethnic nation with 56 officially recognized ethnic minorities, places high importance on the stability and unity of these groups. In minority regions, on one hand, there is a rich and diverse cultural heritage that provides abundant resources for mental health education (Lu, 2012; Wang et al., 2019; Xiao, 2012). On the other hand, there are challenges in providing psychological services in these areas. For example, there is a shortage of mental health professionals, and the local population often shows low enthusiasm, avoidance, or even rejection towards seeking psychological services (Li and Qiang, 2021). The development of mental health perspectives among adolescents in minority regions is relatively lagging, and there is an urgent need for destigmatization education regarding mental health issues (Cheng and He, 2021). Additionally, minority college students often have a non-positive attitude towards mental health services (Wang et al., 2019). Drawing on Guizhou Province as a representative case, the higher education institutions within the province collectively enroll a student body of 881,641 individuals, with 374,043 of these students belonging to ethnic minority groups, which constitutes 42.43% of the total student population (Guizhou Provincial Department of Education, 2022). In Guizhou, there are many challenges in providing mental health services to university students, and the overall mental health level of students is significantly lower than the national average (Wu et al., 2006; Zhang, 2010; Zhang et al., 2008). A survey indicated that the highest ratio of psychological counseling teachers to students in Guizhou universities is only 0.26%. Additionally, about 50% of students feel that the help provided by school counseling centers is minimal or nonexistent, and nearly 30% of students are unwilling to seek counseling voluntarily (Luo, 2012).

In light of the numerous challenges facing mental health education in minority regions, employing peer counselors has emerged as a flexible and promising approach. First, peer counselors can effectively supplement the gaps in professional campus services. Additionally, they are well-equipped to address the challenges posed by multicultural environments. For instance, the unique cultural backgrounds of minority university students can significantly influence their attitudes towards mental illness (Lu, 2012; Tang, 2014). Li highlights the importance of considering local ethnic languages and cultural factors when providing mental health services in minority regions. Incorporating minority personnel who understand ethnic languages and cultures into the composition of mental health service teams can enhance both their effectiveness and acceptance (Li and Qiang, 2021). Minority peer counselors, due to similarities in language and life experiences, are more likely to gain students’ trust. Research shows that minority students tend to prefer peer counselors from their own ethnic groups, as this trust facilitates counseling that is more aligned with their actual needs (Wang and Xie, 2017). Finally, peer counselors play a significant role in addressing the low enthusiasm for seeking mental health services among university students in minority regions. Chen suggests that, given the heightened stigma and shame associated with mental health issues among minority students, schools should encourage student autonomy and create a supportive campus environment for mental health education (Cheng and He, 2021). Sharing positive counseling experiences among peers can effectively reduce the stigma around mental illness and the shame of seeking help (Gronholm et al., 2017; Liu et al., 2017; Ning et al., 2022).

In recent years, there has been a strong call for the development of peer education in mental health education work in the Chinese government (Ministry of Education of the People’s Republic of China, 2021, 2023). In China, the groups engaged in peer education in mental health education include not only class representatives, but also volunteers distributed in dormitories and certain student mental health education clubs, who are collectively known as psychological peer counselors. Psychological peer counselors have become an important role in the current university mental health education in China (Yang et al., 2015). They have contributed to imparting mental health knowledge, organizing regular mental health education campaigns, reporting regularly to mental health education teachers, instructing students to seek professional counseling and effectively identifying and tracking problems (Ma and Ouyang, 2020; Zhan, 2012; Zhang et al., 2009). However, psychological peer counselors, as mental health workers of a semi-professional nature, currently also suffer from a low level of professionalism and a lack of awareness of confidentiality, confusion about their job responsibilities, and unclear role positioning, etc. (Li and Zeng, 2014; Xu, 2009). In order for psychological peer counselors to better assist in advancing mental health education in colleges and universities, it is necessary to explore their competency characteristics.

McClelland first proposed the concept of competency (McClelland, 1973). In 1993 Spencer provided a clear definition of competency traits, which refers to the deep-seated characteristics of an individual that can distinguish the best from the average in a job, including motivation, traits, self-image, attitudes or values, knowledge, cognitive or behavioral skills etc. (Spencer and Spencer, 1993). Spencer believes that there is a causal relationship between competency and career performance. He argues that competencies can be generally described as a set of observable and measurable attributes or success factors required for effective work performance. These attributes or factors may include: knowledge (information a person has in specific content areas), skills (the ability to perform a certain physical or mental task), self-concept and values (a person’s attitudes, values, or self-image), personal traits (physical characteristics and consistent responses to situations or information), and motive (the things a person consistently thinks about or wants), which cause action. Spencer argued that knowledge and skill competencies tend to be visible and relatively surface characteristics of people. Self-concept, trait, and motive competencies are more hidden, deeper, and central to personality. Surface knowledge and skill competencies are relatively easy to develop; training is the most cost-effective way to secure these employee abilities. Core motive and trait competencies are more difficult to assess and develop; it is most cost-effective to select for these characteristics. Establishing criteria for assessing competence is a complex and difficult issue (Kitchener, 2000). In the field of counseling, it requires counselors to have the required knowledge, skills, and abilities and to practice them ethically in order to provide effective services (Barnett and Johnson, 2008). In recent years, research has delved into the competency characteristics of peer psychological counselors in higher education (Hu, 2019; Lai and Liu, 2013; Li, 2021; Li, 2011; Song and Qin, 2017; Su, 2018; Yi, 2014; Zhan et al., 2021; Zhong and Cai, 2014). Lai and Liu suggested a model of peer counselor’s competency characteristics comprising six dimensions: interpersonal communication, self-confidence, conceptual thinking, influence, professional knowledge, and self-control (Lai and Liu, 2013). Similarly, Yi proposed a model for the competency characteristics of psychological committee members, which includes seven dimensions: organizational ability, self-concept, work motivation, teamwork, personality traits, work attitude, and professional knowledge (Yi, 2014). However, an analysis of these models reveals certain limitations in previous research. For instance, these studies do not highlight the importance of professional ethics in the work of psychological counselors. They fail to adopt the perspective of those receiving services, neglecting to thoroughly assess the work requirements of peer psychological counselors. Consequently, this oversight risks blurring the distinctions between the roles of peer psychological counselors and those of typical class student leaders. Another crucial critique is that most of these studies are focused on developed regions in eastern China, with relatively small sample sizes, and overlook the influence of regional and national cultural factors on the competence requirements of psychological service staff. These shortcomings, to some extent, diminish the practical applicability of research on the competency characteristics of peer counseling staff in higher education.

This study aims to leverage Spencer’s competency characteristic theory, utilizing methods such as literature analysis, open-ended questionnaire surveys, and structured interviews. It focuses on understanding the expectations of experts and college students towards the role of peer psychological counselors, as well as the counselors’ self-perception of their functions, in light of the latest developments in their field. Using Guizhou Province as a pilot study, the research comprehensively investigates the competency characteristics of peer psychological counselors in ethnic regions within colleges and universities. A tailored questionnaire assessing these competency characteristics in higher education institutions in ethnic areas will be developed and tested for validity and reliability. The goal is to provide an effective tool for screening, assessing, and evaluating peer psychological counselors in universities and colleges.

## 2 Materials and methods

The development of the Psychological Peer Counselor Competency’s Scale mainly included the construction of the scale item pool, the revision of scale items, exploratory factor analysis, item analysis, and reliability and validity analysis.

### 2.1 Construction of the item pool

#### 2.1.1 Literature review

With the theme of competency of college psychological peer counselors, data related to the competency characteristics of college psychological peer counselors were collected through databases such as CNKI. Additionally, we utilized search tools like SCI-HUB to access relevant research papers. Through merging and organizing the information, a total of 70 terms related to the competency characteristics of college psychological peer counselors were obtained.

#### 2.1.2 Open-ended questionnaires and interviews

##### 2.1.2.1 Interviews with outstanding mental health experts in ethnic regions were conducted

This study engaged mental health education experts from universities in Guizhou Province for interviews. The selection criteria were as follows: (1) holding the position of head at a mental health education center in a university within an ethnic region; (2) over 5 years of experience in the relevant role; (3) prior supervision and management of peer psychological counselors; (4) willingness to participate in the study; and (5) possession of Chinese listening, speaking, reading, and writing skills. We obtained the contact information for these experts through the Mental Health Assistance Platform of Central China Normal University, which recruited mental health experts from nearly a thousand universities nationwide for volunteer services during the COVID-19 pandemic. Invitations were sent to eligible experts, and a total of 15 experts responded to our invitation. Among the participants, there was 1 male and 14 female respondents. The sample included 3 professors, 6 associate professors, and 6 lecturers. The participants had varying years of work experience, ranging from 6 to 26 years. Additionally, 4 participants were supervisors registered in the supervision system of the Clinical and Counseling Psychology Professional Institution of the Chinese Psychological Society, while 3 were psychologists registered in the Clinical and Counseling Psychology Professional Institution of the Chinese Psychological Society. The interview question posed was: What competencies do you consider essential for peer psychological counselors operating within universities in ethnic minority regions? Please articulate the rationale for your perspective. Each expert interview lasted approximately 15–20 min. After the interviews were concluded, the researchers transcribed the audio recordings into text and analyzed the interview data. To enhance the reliability of the analysis, the present study involved two doctoral students in psychology simultaneously analyzing the interview content. The consistency of the coding results between the text coders was examined using categorization consistency and coding reliability coefficients. Category agreement (CA) refers to the percentage of the same coding categorization by the raters for the same interview text, which is calculated as CA = 2 × S/(T1 + T2), where S represents the number of coding categories agreed upon by the raters, T1 is the number of categories coded by Rater 1, and T2 is the number of categories coded by Rater 2 (Martin and Bateson, 1993; Winter, 1994). The formula for the coding reliability coefficient is R = (*n* × average pairwise agreement) / (1 + (*n* − 1) × average pairwise agreement) (Dong, 1992). The average pairwise agreement is calculated as 2 × M/(N1 + N2), where M, N1, and N2 have the same meaning as S, T1, and T2 in the categorization consistency formula. The CA value for the coding of interviews with mental health education experts was 0.70. The coding reliability coefficients was 0.82. Then, the competency feature terms obtained from the categorization consistency test were organized and statistically analyzed. For instance, the term “Respect Privacy” appeared 15 times, which is considered a high-frequency competency feature word and would be retained. Through statistical analysis, a total of 23 competency feature terms for peer counselors were identified.

##### 2.1.2.2 Open-ended questionnaire survey for peer psychological counselors in colleges in ethnic areas

The participants for this survey were recruited from Guizhou Minzu University, Guizhou University, and Guizhou Normal University. With the assistance of the psychological counseling center teachers from these three universities, we randomly selected one peer psychological counselor from each major to distribute the questionnaires. The selection criteria included: (1) willingness to participate in the study; (2) possession of Chinese listening, speaking, reading, and writing abilities; (3) at least 0.5 years of experience working as a peer psychological counselor. During the after-class time, the research team members invited students to participate in the study. Students were informed that they had three days to consider whether to participate in this research. Those willing to participate in the study would submit a complete survey questionnaire, while those who refused to participate were asked to return the blank survey questionnaire. Research assistants assigned numbers to the students and anonymized the data to protect their privacy. To avoid putting pressure on the students, the lead researcher was unaware of which students had participated and which had not. A total of 50 questionnaires were distributed, and 48 were collected (15 male, 33 female). Among the respondents, 21 were freshmen, 15 were sophomores, 8 were juniors, and 4 were seniors. Furthermore, 23 participants belonged to ethnic minorities, while 25 were from the Han Chinese ethnicity. The open-ended questionnaire consisted of four questions: 1. How do you feel about your current work in peer counseling? 2. Do you consider yourself competent in this role? Why or why not? 3. What abilities or qualities do you believe are necessary for effective performance in this role? 4. In our ethnic area, how can we adapt our counseling approach to meet the needs of students from diverse ethnic backgrounds? The responses to the open-ended questionnaire survey were analyzed and processed. Similarly, two doctoral students in psychology analyzed the content of the open-ended questionnaire, and the consistency of the coding results between text coders was examined using CA and coding reliability coefficients. The CA value for the open-ended questionnaire results was 0.70, and the coding reliability coefficient was 0.82. After extracting words related to the competency characteristics of psychological peer counselors in colleges and universities, a total of 24 words were identified.

##### 2.1.2.3 Individual and group interviews were with college psychological peer counselors

The participants for individual and group interviews were recruited through advertisements at Guizhou Minzu University and Guizhou University. The selection criteria included: (1) willingness to participate in the study; (2) possession of Chinese listening, speaking, reading, and writing abilities; and (3) at least 0.5 years of experience working as a peer psychological counselor. For individual interviews, a total of 12 participants were interviewed, comprising 4 males and 8 females. Among them, 5 were freshmen, 4 were sophomores, 2 were juniors, and 1 was a senior. Additionally, 6 participants belonged to ethnic minorities, while the remaining 6 were Han Chinese. Each individual interview lasted approximately 30 min, and the questions posed during the interviews were as follows: (1) How do you feel about your current involvement in peer counseling work? (2) What has been the most challenging experience you have faced in your role? (3) What competencies or qualities do you believe are essential for effectively performing the duties of a peer psychological counselor? Please provide specific examples and detailed reasons to support your answer. (4) As a peer psychological counselor, what feedback or suggestions have you received from your supervisory teachers or peers? In addition, there were a total of 4 group interviews, each with 6 participants, totaling 24 individuals. Among them, 9 participants were male and 15 were female. 10 participants were freshmen, 8 were sophomores, 4 were juniors, and 2 were seniors. Moreover, 11 participants belonged to ethnic minorities, while 13 were Han Chinese. A total of four interview groups were recruited, each consisting of six individuals, amounting to 24 participants in total. Among them, there were 9 males and 15 females; 10 were freshmen, 8 were sophomores, 4 were juniors, and 2 were seniors; 11 were from ethnic minorities, and 13 were Han Chinese. Each group interview lasted between 50 to 60 min, and the main content of the interviews included the following questions: (1) How did you become a peer psychological counselor, and what are your feelings about this role? (2) What competencies or qualities do you think are necessary to excel as a peer psychological counselor? (3) What challenges have you encountered in your work, and how have you addressed them? As a token of appreciation, each participant was presented with a gift valued at 50 yuan RMB following the completion of the interview. The processes of individual and group interviews were both recorded. Two doctoral students in psychology analyzed the text transcribed from the recordings. Similarly, the consistency of the coding results between text coders was examined using CA and coding reliability coefficients. The CA value for the extraction of content feature words from individual interviews was 0.74, with a coding reliability coefficient of 0.85. The CA value for the extraction of content feature words from group interviews was 0.71, with a coding reliability coefficient of 0.83. By delving into the data obtained from both individual and group interviews, we identified a total of 23 distinctive keywords that are linked to the competencies and characteristics of peer psychological counselors.

##### 2.1.2.4 Interviews with excellent peer psychological counselors

To understand the competency characteristics of outstanding peer psychological counselors, we conducted interviews with peer psychological counselors who have been recognized as excellent by the superior administrative authorities. We recruited the interviewees through recommendations from the heads of the school mental health education centers at 10 universities in Guizhou Province. These universities are: Guizhou Minzu University, Guizhou University, Guizhou Normal University, Guizhou Medical University, Guizhou University of Finance and Economics, Guizhou University of Traditional Chinese Medicine, Guizhou Light Industry University, Guizhou Communication Polytechnic College, Guizhou Light Industry Vocational College, and Guiyang Police College. The selection criteria included: (1) willingness to participate in the study; (2) possession of Chinese listening, speaking, reading, and writing abilities; (3) at least 0.5 years of experience working as a peer psychological counselor; and (4) had been awarded honors or titles for excellence as peer psychological counselors at the institutional level or higher. We recruited one interviewee from each university, totaling 10 outstanding peer psychological counselors who participated in our interviews. Among them, there is 1 National Top 100 Psychological Committee member, 1 National Top 100 Psychological Committee member candidate, 8 school-level excellent peer psychological counselors; 2 male and 8 female; 3 sophomores, 5 juniors, and 2 seniors; 5 ethnic minorities and 5 Han Chinese. The interview questions included: (1) Why do you want to become a peer psychological counselor? (2) How do you feel about your current engagement in peer counseling work? (3) What abilities or qualities do you believe an excellent peer psychological counselor should possess? Please provide detailed examples to illustrate. (4) Please enumerate an event in your work that you found most satisfying, and provide as detailed and specific a description as possible. (5) Please enumerate an event in your work that you found most regrettable, and provide as detailed and specific a description as possible. Each participant’s interview lasted approximately 40 min. At the end of the interview, each participant received a souvenir worth 50 Chinese yuan as a token of appreciation. The interview process was recorded, and two doctoral students in psychology analyzed the text transcribed from the recordings. Similarly, the consistency of the coding results between text coders was examined using CA and coding reliability coefficients. The CA value for the extraction of content feature words from interviews with excellent peer psychological counselors was 0.73, and the categorization agreement coefficient was 0.84. After the interviews were generalized and processed, and after extracting the words related to the competency characteristics of the peer psychological counselors in colleges and universities, 23 competency characteristic words were obtained.

##### 2.1.2.5 Interviews with students who have received help from peer counselors

To explore the competency profile of peer psychological counselors as perceived by those they assist, we initiated a series of interviews with students who had availed themselves of peer counseling services at Guizhou Minzu University, reaching out through online advertisements. The criteria for selecting these interviewees were stringent, encompassing: (1) having accumulated more than 10 h of peer counseling services, which entailed activities such as attending mental health awareness seminars, group counseling sessions, and individual consultations; (2) a willingness to engage in the study; and (3) proficiency in Chinese language skills, including listening, speaking, reading, and writing. Among them were 1 male and 19 female participants. Their academic levels consisted of 6 freshmen, 8 sophomores, 2 juniors, and 4 seniors. Furthermore, 11 participants belonged to ethnic minorities, while 9 participants were Han Chinese. The interview questions were as follows: (1) Drawing on your experiences and personal feelings, what abilities or qualities do you believe a peer psychological counselor should have? (2) Were you satisfied with the peer psychological counselors who have served you? Please provide a detailed explanation of your reasons. Each participant’s interview lasted approximately 30 min. As a token of appreciation, each participant was presented with a gift valued at 100 yuan RMB following the completion of the interview. After the interviews concluded, two doctoral students in psychology analyzed the transcribed text from the recordings. Likewise, the reliability of the text coding was assessed using CA and the categorization agreement coefficients. For the extraction of content feature words from the interviews with college students who were served by peer psychological counselors, the CA value stood at 0.70, with a coding reliability coefficient of 0.82. The interviews underwent inductive processing to generalize the content, and relevant words and phrases related to the competence of psychological peer counselors in colleges and universities were extracted. A total of 23 vocabulary terms describing the competence characteristics of psychological peer counselors in colleges and universities were obtained.

### 2.2 Questionnaire distribution and data collection

The questionnaires were distributed in paper format in two installments. A random stratified sampling method was used to select the participants, with psychological peer counselors from two randomly chosen majors at the following 12 universities in Guizhou Province: Guizhou University, Guizhou Normal University, Guizhou Medical University, Guizhou University of Traditional Chinese Medicine, Guizhou Communication Polytechnic College, Zunyi Medical University, Anshun College, Guiyang Police College, Guizhou Light Industry University, Guizhou University of Finance and Economics, Guizhou Minzu University, and Guizhou Light Industry Vocational College. The types of these universities cover comprehensive, normal, engineering, and agricultural/medical disciplines. The criteria for participant selection were as follows: (1) willingness to participate in the study; (2) willingness to sign the consent form; (3) possession of Chinese listening, speaking, reading, and writing abilities; and (4) at least 0.5 years of experience working as a peer psychological counselor. The first data collection period was from March 15 to March 21, 2022 (Sample A). The preliminary measurement scale was administered to participants. We distributed a total of 460 questionnaires, and out of those, we received 450 valid responses. Among the respondents, there were 92 male students and 358 female students. The distribution among academic years was as follows: 256 freshmen, 122 sophomores, 63 juniors, 6 seniors, and 3 graduate students. Additionally, there were 238 Han Chinese students and 238 ethnic minority students. The age range of the participants was between 17 and 30, with an average age of (20.40 ± 1.676) years. The data from this period were used for exploratory factor analysis (EFA) and item analysis. Additionally, we selected 230 psychological peer counselors from our sample for retesting at 3-month intervals. The retesting group included 49 males and 181 females. Among them, there were 138 freshmen, 28 sophomores, 29 juniors, 2 seniors, and 3 graduate students. The second data collection phase was from March 22 to March 28, 2022 (Sample B). 600 questionnaires were distributed, and 579 valid questionnaires were collected. Among the respondents, there were 145 male students and 434 female students. The distribution across academic years included 259 freshmen, 174 sophomores, 107 juniors, 35 seniors, and 4 postgraduates. Additionally, there were 258 Han Chinese students and 321 ethnic minority students. The participants’ ages ranged from 17 to 30 years old, with an average age of (20.43 ± 1.717) years old. The revised scale based on EFA was distributed to collect data for verifying the reliability and validity of the scale.

### 2.3 Criterion

#### 2.3.1 The interpersonal reactivity index

The Interpersonal Response Index (ICR-R) is an instrument developed by Davis to measure empathy based on the multidimensional theoretical construct of empathy (Davis, 1980). The Chinese version of the Interpersonal Reactivity Index (IRI-R) is a 22-item scale that measures four dimensions: Perspective Taking, Fantasy, Empathic Concern, and Personal Distress (Zhang et al., 2010). The scale employs a Likert five-point rating system, ranging from 0 to 4, with response options varying from “totally inappropriate” to “completely appropriate.” In the present study, the Cronbach’s alpha coefficient for this scale was calculated to be 0.70.

#### 2.3.2 The general self-efficacy scale

The Chinese version of the General Self-Efficacy Scale (GSES) was revised by Zhang and Schwarzer (1995). The GSES is a 10-item scale designed to assess overall self-efficacy. Responses are recorded on a four-point scale ranging from “very poorly (1)” to “very well (4).” The total self-efficacy score is obtained by summing up the scores for all the questions, with higher scores indicating a stronger sense of self-efficacy. In this study, the Cronbach’s alpha coefficient for this scale was 0.86.

### 2.4 Data analysis

In this study, we employed SPSS 24.0 for exploratory factor analysis (EFA) and MPLUS 8.0 for confirmatory factor analysis (CFA). Initially, we performed EFA and item analysis on the predictive sample (*n* = 450). Subsequently, CFA, validity scale validity testing, and internal consistency testing were conducted on the formal administration sample (*n* = 579). Finally, retest reliability analysis was carried out on the retest sample (*n* = 230).

Before the study, informed consent was acquired from the participants. Furthermore, the research protocol received approval from the Institutional Review Board of the University. All activities carried out during the study involving human subjects were conducted in strict adherence to the ethical guidelines outlined by the Institutional Review Board of the University, as well as in accordance with the principles set forth in the 1964 Declaration of Helsinki and its subsequent revisions, or equivalent ethical benchmarks.

## 3 Results

### 3.1 Exploratory factor analysis

The KMO test and Bartlett’s test of sphericity were conducted to examine whether the variables were suitable for factor analysis. The KMO value obtained was 0.933, and the *p*-value was <0.001, indicating that the data met the requirements for factor analysis. The principal component analysis with varimax rotation was used to extract common factors, based on the criterion of eigenvalues greater than 1, combined with the scree plot to determine the number of factors. Items were then removed based on the magnitude of the communalities and factor loadings. Firstly, items with factor loadings less than 0.50 and communalities less than 0.20 were deleted. Then, items with fewer than three on the same factor were removed. After multiple rounds of exploratory factor analysis, items 1, 4, 6, 9, 12, 13, 14, 15, 17, 23, 24, 27, 33, 35 and 36 were deleted, forming a scale with 21 items and 4 factors. The cumulative variance contribution rate of the factors was 66.26%. Factor 1, composed of 8 items, accounted for 21.94% of the variance. Factor 2, composed of 5 items, accounted for 18.33% of the variance. Factor 3, composed of 4 items, accounted for 13.96% of the variance. Factor 4, composed of 4 items, accounted for 12.02% of the variance. According to the meaning of the items in each factor group, the factors were defined as factor 1 for Role Identity, factor 2 for Communication Sensitivity, factor 3 for Personal Traits and factor 4 for Professional Ethics (see Table 1 for details).

**Table 1:** Exploratory factor analysis of the psychological peer counselor competency’s scale (*n* = 450).

Factor 1		Factor 2		Factor 3		Factor 4	
Item	Coefficient of factor load	Item	Coefficient of factor load	Item	Coefficient of factor load	Item	Coefficient of factor load
11	0.79	16	0.79	28	0.81	10	0.73
29	0.72	25	0.77	32	0.68	5	0.71
19	0.69	21	0.72	8	0.65	3	0.59
22	0.66	31	0.64	26	0.57	34	0.55
2	0.62	30	0.60				
18	0.59						
7	0.57						
20	0.57						

### 3.2 Item analysis

Subjects in the upper and lower 27% of the total score in the overall sample were selected to be divided into high and low subgroups. An independent sample *t*-test between the two groups was conducted and found that there were very significant differences between the two groups in the scores of each of the 21 items (*t* = 7.43–20.99, all *p* < 0.001). The results indicate that each item had a high degree of differentiation and could distinguish the two groups significantly. Further analysis of the correlation between each item and the total score found that there was a very significant correlation between the scores of each of the 21 items and the total score, and all the correlation coefficients were greater than 0.5(*r* = 0.51–0.78, all *p* < 0.01). This shows that the items in this scale were highly correlated with competence.

### 3.3 Reliability and validity analysis of the psychological peer counselors’ competence scale

#### 3.3.1 Reliability analysis of the scale

##### 3.3.1.1 Internal consistency reliability

The Cronbach’s alpha value of the total score was 0. 95 and The Cronbach’s alpha value of the factors of Role Identity, Communication Sensitivity, Personal Traits, and Professional Ethics were 0.95, 0.88, 0.84, and 0. 73, respectively.

##### 3.3.1.2 Test–retest reliability

The correlation coefficient between the total scores of the two scales was found to be 0.92, and the correlation coefficient for the factors of Role Identity, Communication Sensitivity, Personal Traits, and Professional Ethics were 0.72, 0.88, 0.85, and 0.64, respectively.

##### 3.3.1.3 Split-half reliability

The Spearman-Brown coefficient of the total score of the scale was 0.94, and The Spearman-Brown coefficient of the factors of Role Identity, Communication Sensitivity, Personal Traits, and Professional Ethics were 0. 89, 0.85, 0.85, and 0.71, respectively.

The scale can thus be considered to have good reliability.

#### 3.3.2 Validity analysis of the scale

##### 3.3.2.1 Confirmatory factor analysis

Based on the results of the EFA, the structure of the four factors was validated using Mplus8.0, and the results are shown in Table 2. The ratio of chi-square to degrees of freedom, SRMR, TLI/NNFI, CFI, RMSEA, and other indicators of model fitting obtained by CFA are presented in Table 2. Obviously, all the indicators were in line with the criteria. This shows that the scale fit the model well.

**Table 2:** Model fit indicators for the psychological peer counselor competency’s scale.

Criteria	*X^2^/df*	*SRMR*	*TLI/NNFI*	*CFI*	*RMSEA*	*AIC*	*BIC*
Value	2.67	0.04	0.93	0.94	0.05	21960.17	22261.10

##### 3.3.2.2 Criterion validity

The total score and factor scores of the Competency Scale for Psychological peer counselors in Colleges in Ethnic Areas (*n* = 579) were positively correlated with the IRC-C total score (83.02 ± 6.17) and the GSES total score (27.76 ± 3.46) (Table 3).

**Table 3:** Correlation of the psychological peer counselor competency’s scale with validity scales.

Item	*M ±* SD	ICR-R	GSES
Role identity	30.42 ± 5.11	0.22**	0.62**
Communication sensitivity	20.41 ± 3.03	0.19**	0.57**
Personal traits	15.80 ± 2.25	0.21**	0.58**
Professional ethics	17.25 ± 2.35	0.18**	0.51**
Total score	83.90 ± 11.68	0.23**	0.64**

** *p* < 0.01.

## 4 Discussion

This study constructs a scale for peer psychological counselors in multi-ethnic university contexts, conforming to the rigorous standards of psychological measurement. The development process began with the establishment of an item pool, which was informed by a comprehensive literature review, expert interviews, open-ended questionnaires, individual and group interviews with peer psychological counselors, interviews with outstanding peer psychological counselors, and feedback from university students who had previously received their services. Building upon Spencer’s competency theory, the item pool was meticulously refined through an integration of literature analysis, open-ended questionnaire responses, and interview data. This process culminated in a preliminary questionnaire featuring 36 items designed to assess the competencies of peer psychological counselors within university settings. In the subsequent exploratory factor analysis, items 1, 4, 6, 9, 12, 13, 14, 15, 17, 23, 24, 27, 33, 35, and 36 were identified for removal, while the item analysis phase did not necessitate the exclusion of any items. This refinement led to the creation of a formal questionnaire structured around four distinct factors. The confirmatory factor analysis conducted for this study yielded favorable fit indices, confirming the model’s adequacy. The scale developed in this research demonstrated strong criterion validity when compared to the ICR-R scale and the GSES. Furthermore, reliability analysis results indicated that the questionnaire met the psychometric standards for internal consistency reliability, test–retest reliability, and split-half reliability.

This study found that the competency structure of psychological peer counselors in colleges in multi-ethnic regions contains four factors: role identity, communication sensitivity, personal traits, and professional ethic.

Role identification refers to the intrinsic meaning associated with a specific role and the expectations that people have for that role. Therefore, individuals can assume corresponding roles based on the description of role identification (Yan et al., 2011). In this study, the relevant items related to role identification primarily involve the need for peer counselors to possess relevant professional knowledge, skills, and work attitude. Similar conclusions were also found in the study conducted by Zhan et al. (2021) regarding class psychological committee members (Zhan et al., 2021). Both professional knowledge and skills, as well as work attitude, are requirements and core aspects of the role of peer counselors. They are closely related to the positioning and requirements of the role, leading to a significant intersection between these two situations and a high correlation. In the exploratory factor analysis, they form a common factor, which is thus collectively referred to as role identification. In previous studies on the competence of psychological peer counselors, specialized knowledge has been viewed as an independent dimension, while the role identity of psychological peer counselors has not been mentioned (Lai and Liu, 2013; Yi, 2014). However, role identification specifies the expectations others have for psychological peer counselors, and a sense of self-identity among psychological peer counselors is significantly positively correlated with their motivation and subjective initiative in task completion, enabling them to better fulfill their peer counseling responsibilities.

An important finding of this study is the factor of communication sensitivity, which includes not only communication skills but also multicultural competence. Previous domestic investigations into the competence structure of psychological peer counselors predominantly emphasized the significance of communication skills (Hu, 2019; Lai and Liu, 2013; Li et al., 2011; Li, 2021; Zhan et al., 2021), aligning with the findings of our study in this context. However, to the best of our knowledge, this study is the first empirical study to demonstrate the impact of multicultural competence on the competence of psychological peer counselors in ethnic areas of China (Aggarwal et al., 2016; Kleinman et al., 2016; Nanda and Warms, 2021; Yeung et al., 2010). Sue defines cultural competence as the ability to “act or create actions that are most suitable for the development and systemic situation of the client,” believing that individual cultural competence should involve awareness, knowledge, and skills to effectively apply in a diverse society. In terms of organizational cultural competence, it should be able to advocate and develop new theories, practices, and policies to effectively respond to diverse cultural groups (Constantine and Sue, 2006; Sue, 2005). Cultural competence in mental health services refers to a series of capabilities of service providers (such as counselors or therapists, sometimes also referring to service organizations or institutions) to address cultural issues, enabling service providers to intervene more effectively with visitors from diverse cultural groups (Sue et al., 2009). Currently, various countries have established training systems for cultural competence based on their needs (American Psychological Association, 2003, 2015; Clauss-Ehlers et al., 2019; Kirmayer, 2012; Schouler-Ocak et al., 2015). In China, Wang Ming and his colleagues propose that the core competencies of psychotherapists should encompass six aspects: professional attitude and behavior, ethical and legal knowledge, clinical knowledge and skills, scientific research, interpersonal relationship skills, multicultural and Chinese cultural awareness, and case management (Wang et al., 2015). For psychological peer counselors in ethnic universities, communicating with help-seekers from diverse ethnic and cultural backgrounds, being aware and understanding the potential impact of different cultural backgrounds on the values and behaviors of help-seekers, and adjusting their work methods are crucial for effective work.

Personal traits are gradually formed by individuals in long-term social life, representing a stable attitude towards the real world and habitual behavior patterns. These traits exhibit relative stability in different periods and environments. Gilbert et al. argue that appropriate personal traits are crucial and fundamental for individuals engaged in psychological counseling and therapy to succeed in their work (Gilbert et al., 2014). Hence, numerous studies, both domestic and international, on the competency characteristics of mental health service providers emphasize the importance of their personal traits. For instance, Menne identified 12 dimensions of counselor competency characteristics through factor analysis, with personal traits ranking first in terms of the explained variance (Menne, 1975). In Roe’s “Competency Characteristics Construction Model” for mental health service providers, abilities, personality traits, and other personal characteristics are considered the foundation of the model (Roe, 2002). Some domestic studies on the competency characteristics of mental health service providers also to varying degrees underscore the importance of their personal traits (Liang, 2008; Xie, 2008). Su et al. suggest that personal traits should be emphasized in the selection of psychological peer counselors (Hu, 2019; Li, 2011; Song and Qin, 2017; Su, 2018; Wang, 2014). Given the unique nature of the work of psychological peer counselors, traits such as a healthy psychological condition, the ability to build positive relationships with others, influence, and a genuine concern for others are crucial for the smooth implementation of peer mental health education. Therefore, personal traits have a certain screening nature and can help identify individuals better suited for the role of psychological peer counselors.

The requirements of professional ethics factors include fairness, respect, and confidentiality towards help-seekers. In the early studies on the competency characteristics of mental health service providers in foreign countries, researchers identified “professional ethics” as a component of counselor competency characteristics through factor analysis (Menne, 1975). At the Competency Characteristics of Mental Health Service Providers Conference held in the United States in 2002, participants unanimously agreed that having professional ethics is an important competency characteristic of mental health service providers (Kaslow et al., 2004). Zhang et al. pointed out that professional ethics is one of the core competency characteristics of mental health service providers (Zhang and Huang, 2014). The “Chinese Psychological Association Code of Ethics for Clinical and Counseling Psychology” mentions virtues, responsibility, integrity, fairness, and respect (Chinese Psychological association, 2018). In previous empirical studies exploring the competency characteristics of psychological peer counselors in domestic universities, only Zhan et al. confirmed that ethical factors are an important part of the job competence of psychological peer counselors (Zhan et al., 2021). In actual work, adhering to professional ethics is of utmost importance for psychological peer counselors. Li et al. found that a lack of attention to ethics among psychological peer counselors can cause more severe psychological burdens on students with psychological issues, making it more difficult to carry out school psychological crisis work (Li and Zeng, 2014). The inclusion of “professional ethics” as a factor in the structure of competency characteristics of psychological peer counselors in universities not only has theoretical rationality but also holds important practical significance.

During the development process of the scale, we also encountered several challenges. For instance, unlike previous studies that developed competency scales for mental health service providers, this research introduced surveys of university students who had been served by peer psychological counselors during the qualitative analysis phase (Hu, 2019; Li, 2021; Zhan et al., 2021). This addition aimed to help us understand the needs of the service recipients regarding the competencies of peer psychological counselors. It took a considerable amount of time to recruit college students who had been served by peer counselors, with the stigma and shame associated with mental health issues likely being a major factor. We ultimately this barrier by modifying the content of the recruitment advertisement, such as emphasizing the purpose of the study and the confidentiality, increasing the value of the incentives, and expanding the distribution scope of the advertisements. The analysis of the interview and open-ended questionnaire data was conducted independently by two PhDs in psychology. We provided training for the coders in advance and used CA to verify the reliability of the coding categories.

The study has several limitations. Firstly, the questionnaire was administered exclusively in Guizhou Province, meaning the selected items reflect the competency characteristics of peer psychological counselors specifically among university students in this region. As a result, cultural differences related to ethnicity and locality should be carefully considered when applying this scale to other minority regions. Additionally, the study did not include further applied exploration and analysis, which should be a primary focus for future research. In the future, data from nine other minority regions in China will be gathered to conduct a measurement invariance study using this scale. As the scale becomes more widely utilized and more samples are accumulated, it will contribute to establishing a more stable and reliable competency standard for peer psychological counselors in universities across ethnic minority regions. Beyond its use in selection processes, the scale developed in this study holds significant practical value. For example, it can be used for researching training programs for peer psychological counselor competencies, evaluating the effectiveness of such training, and designing targeted interventions for areas where competency is lacking. Furthermore, longitudinal follow-up studies could be conducted to analyze the developmental trajectories of peer psychological counselor competencies in ethnic minority regions and to explore variables that may influence these competencies.

## 5 Conclusion

The competency scale developed in this study for psychological peer counselors in universities in ethnic regions exhibits good reliability and validity. This scale is suitable for screening and assessing the abilities of psychological peer counselors in multi-ethnic universities.

## Data Availability

The raw data supporting the conclusions of this article will be made available by the authors, without undue reservation.
