# Supplementary material for: Developing a competency scale for selecting and assessing psychological peer counselors in ethnic-area colleges: a pilot study in Guizhou Province, China
Source: Front Psychol. 2024 Dec 11;15:1402403. doi: 10.3389/fpsyg.2024.1402403 (PMC11669043; doi:10.3389/fpsyg.2024.1402403)
Supplement: Supplementary file 1 [file Data_Sheet_1.docx]

**Competency Questionnaire for Peer Counselors in Colleges and Universities in Ethnic Areas**

| question | 1 | 2 | 3 | 4 | 5 |
| --- | --- | --- | --- | --- | --- |
| 1.I am very passionate about the work of peer psychological counselors. |  |  |  |  |  |
| 2.In my work, I respect and strive to understand the behaviors and values of classmates from different cultural backgrounds. (Different cultural backgrounds include: different genders, ages, gender identities, ethnicities, cultures, religions, sexual orientations, languages, socioeconomic statuses, etc.) |  |  |  |  |  |
| 3.I can treat each classmate fairly and justly. |  |  |  |  |  |
| 4.I have a certain understanding of knowledge related to maintaining mental health. |  |  |  |  |  |
| 5. I can bring positive and uplifting influence to others, often described as having a "positive energy." |  |  |  |  |  |
| 6.I will not casually comment on the privacy of classmates. |  |  |  |  |  |
| 7.I am familiar with my responsibilities and the relevant work processes in school mental health education. |  |  |  |  |  |
| 8.During daily interactions, I excel at detecting changes in others' emotions. |  |  |  |  |  |
| 9.I can promptly identify classmates experiencing psychological crises. |  |  |  |  |  |
| 10.I aspire to be proficient in the role of a peer psychological counselor. |  |  |  |  |  |
| 11.I demonstrate a robust commitment to collaborative teamwork. |  |  |  |  |  |
| 12.I will strive to study and understand the potential impact of different cultural backgrounds on individual behaviors and values, enabling me to better comprehend my classmates. (Different cultural backgrounds include: different genders, ages, gender identities, ethnicities, cultures, religions, sexual orientations, languages, socioeconomic statuses, etc.) |  |  |  |  |  |
| 13.I can actively and proactively accomplish the mental health education-related tasks assigned by the school. |  |  |  |  |  |
| 14.When communicating with classmates from different cultural backgrounds, I can perceive the impact that these backgrounds have on them. (Different cultural backgrounds include: different genders, ages, gender identities, ethnicities, cultures, religions, sexual orientations, languages, socioeconomic statuses, etc.) |  |  |  |  |  |
| 15.I can try my best to care for every classmate. |  |  |  |  |  |
| 16.I can regulate my negative emotions and maintain a positive mindset. |  |  |  |  |  |
| 17.I can organize and implement various psychological resilience-building activities for college students. |  |  |  |  |  |
| 18.When communicating with classmates from diverse cultural backgrounds, I conscientiously adjust my working methods to achieve better outcomes. (Diverse cultural backgrounds encompass various factors such as different genders, ages, gender identities, ethnicities, cultures, religions, sexual orientations, languages, socioeconomic statuses, etc.) |  |  |  |  |  |
| 19.I have a strong sense of empathy. |  |  |  |  |  |
| 20.I can easily establish relationships with others and earn their trust. |  |  |  |  |  |
| 21.I will not disclose the situations of classmates to anyone, except in cases of confidentiality exceptions, such as when a classmate expresses intentions to harm themselves or others. |  |  |  |  |  |

**Note：1= Particularly incompatible；2 =relative lack of conformity；3= Inconclusive；4= relatively compatible；5=particularly compatible**
